# Factors Associated with Secondary Pulmonary Hypertension Among Hospitalized Females: An Artificial Neural Network Analysis of a National US Cohort

**DOI:** 10.3390/jpm16080421

**Published:** 2026-08-07

**Authors:** Adil Sarvar Mohammed, Sai Priyanka Mellacheruvu, Zainab Gandhi, Sai Prasanna Lekkala, Suvidha Manne, Umera Yasmeen, Iramunisa Begum, Rupak Desai, Shrinivas Kambali, Lakshmi Sai Meghana Kodali, Shiny Teja Kolli, Shaylika Chauhan, Shweta Kambali

**Affiliations:** 1 Department of Internal Medicine, Covenant HealthCare College of Medicine at Central Michigan University, Saginaw, MI 48602, USA; moham2as@cmich.edu; 2 Department of Internal Medicine, Trinity Health-Nazareth Hospital, Philadelphia, PA 19152, USA; saipriyanka.mellacheruvu@mercyhealth.org; 3 Department of Pulmonary and Critical Care Medicine, Cleveland Clinic, Cleveland, OH 44195, USA; gandhiz@ccf.org; 4 Department of Internal Medicine, UCHealth Parkview Medical Center, Pueblo, CO 81003, USA; saiprasanna.lekkala@uchealth.org; 5 Department of Internal Medicine, St. Luke’s Medical Center, Chesterfield, MO 63017, USA; suvidha.manne@stlukes-stl.com; 6 Department of Internal Medicine, Mamata Medical College, Khamma 507002, India; umera.uy@gmail.com; 7 Department of Internal Medicine, Shadan Institute of Medical Sciences, Hyderabad 500008, India; iramulous@gmail.com; 8 Independent Researcher, Atlanta, GA 30033, USA; drrupakdesai@gmail.com; 9 Department of Pulmonary and Critical Care Medicine, MyMichigan Medical Center, Saginaw, MI 48601, USA; shrinivas.kambali@mymichigan.org; 10 Department of Public Health and Health Sciences, University of Michigan-Flint, Flint, MI 48502, USA; 11 Department of Internal Medicine, Phoebe Putney Memorial Hospital, Albany, GA 31701, USA; stkolli@phoebehealth.com; 12 Department of Internal Medicine, Geisinger Health System, Wilkes-Barre, PA 18711, USA; schauhan@geisinger.edu; 13 Department of Internal Medicine, McLaren Bay Region, Bay City, MI 48708, USA; shweta.kambali@mclaren.org

**Keywords:** non-group-1 pulmonary hypertension, pulmonary arterial hypertension, secondary pulmonary hypertension, females/women, predictors/risk factors, outcomes/mortality

## Abstract

**Background**: Non-group 1 pulmonary hypertension, also known as secondary pulmonary hypertension (SPH), is predominantly observed among females. However, there is a significant lack of data concerning factors associated with hospitalization among patients diagnosed with SPH. This study aims to provide clinicians with vital insights for the identification of high-risk groups and for the more effective management of contributory risk factors within the female population affected by SPH. **Methods**: Using the 2019 National Inpatient Sample, we identified female admissions with SPH (n = 648,190), accounting for 3.8% of the total 17,236,228 female admissions. An Artificial Neural Network (ANN) analysis was conducted to evaluate predictive factors. We randomly allocated 3,319,543 patients into training and testing datasets at a ratio of 70:30, comprising 2,323,696 (70%) for training and 995,847 (30%) for testing, to calibrate and validate the performance of the ANN algorithm. Model performance was assessed by comparing misclassification rates between training and testing sets and by the area under the receiver operating characteristic curve (AUC); only internal validation was performed. **Results**: Females hospitalized with SPH were generally of older age, with a median of 75 years compared to 58 years, and more frequently identified as White (67.7% versus 65.5%) or Black (20.5% versus 15.5%) relative to those without SPH. They also demonstrated a higher prevalence of most atherosclerotic cardiovascular disease (ASCVD) risk factors or their equivalents, including complicated hypertension (50.6% versus 17.8%), diabetes with chronic complications (30.6% versus 13.7%), and hyperlipidemia (50.8% versus 29.2%), as well as other comorbidities such as COPD (43.4% versus 20.2%) and CKD (43.3% versus 14.0%), and exhibited increased all-cause mortality (4.5% versus 1.8%) (*p* < 0.001). Our ANN model achieved an AUC of 0.823, indicating good predictive capability. The rates of incorrect predictions were comparable in both the testing and training cohorts, at 3.8% each. The factors most strongly associated with a coded SPH diagnosis included age at admission, complicated hypertension, chronic kidney disease, chronic obstructive pulmonary disease, uncomplicated hypertension, prior VTE, race, arthropathies, and AIDS. **Conclusions**: Our ANN model identified demographic and comorbidity factors associated with a coded SPH diagnosis among hospitalized females, with good discrimination (AUC = 0.823). Because the model classifies the presence of an existing diagnosis rather than predicting future hospitalization, and was validated only internally, external and prospective validation is required before clinical application. Once validated, these factors could support individualized, sex-specific risk stratification for high-risk female populations, consistent with the goals of personalized medicine.

## 1. Introduction

Pulmonary hypertension (PH) is a hemodynamic condition defined by a resting mean pulmonary arterial pressure (mPAP) exceeding 20 mmHg, in accordance with the 2022 European Society of Cardiology/European Respiratory Society (ESC/ERS) guidelines. These guidelines revised the previous threshold of ≥25 mmHg to better reflect the upper limit of normal pulmonary arterial pressure (PAP) and its prognostic significance [1]. PH is classified into five groups: Group 1 (pulmonary arterial hypertension) results from primary pulmonary vascular disease, whereas non-Group 1, or secondary pulmonary hypertension (SPH), includes Group 2 (left heart disease), Group 3 (lung disease and/or hypoxia), Group 4 (chronic thromboembolic pulmonary hypertension and other pulmonary artery obstructions), and Group 5 (unclear and/or multifactorial mechanisms) [1]. Due to substantial differences in pathophysiology, prognosis, and treatment among these groups, SPH should not be regarded as a singular clinical entity. A female predominance in PH has been reported across various decades and PH subtypes. National surveillance data from the United States covering 2001 to 2010 indicated that PH-related hospitalization and mortality rates consistently exceeded those of men, with the female-to-male ratio increasing over the decade [2]. Nonetheless, comprehensive, large-scale data concerning the demographic and comorbidity factors associated with SPH among hospitalized women remain limited. This underscores the importance of identifying clinical characteristics linked to SPH-related hospitalizations, which could enable earlier detection of high-risk women and support personalized, sex-specific management, a fundamental objective of personalized medicine. Accordingly, we conducted an analysis using the National Inpatient Sample, combined with an Artificial Neural Network (ANN), a machine learning algorithm capable of processing extensive datasets and detecting subtle, non-linear patterns within complex health data.

## 2. Methods

This population-based cross-sectional study employed retrospective data from the 2019 National Inpatient Sample [3] to investigate hospitalizations of adult females carrying a diagnosis of secondary (non-group 1) pulmonary hypertension (SPH), identified using the International Classification of Diseases, 10th Revision, Clinical Modification (ICD-10-CM) diagnostic code I27.2x. Because the analysis compares hospitalized women with and without this code, it characterizes factors associated with the presence of a coded SPH diagnosis at the encounter level rather than predicting future hospitalization among patients with established SPH. Categorical variables were analyzed using Pearson’s Chi-square test, while continuous variables, which did not follow a normal distribution, were compared using the Mann–Whitney U test. A *p*-value of less than 0.05 was considered indicative of statistical significance.

Inclusion and exclusion criteria: The study population comprised adult female inpatient discharges in the 2019 National Inpatient Sample (NIS). Records were assigned to the SPH group if they carried an I27.2x (secondary/non-group 1 PH) code, and to the comparison group if they did not. Records with missing values on one or more model variables were excluded from the neural-network analysis (127,703 of 3,447,246 unweighted female discharge records; see below). Congenital heart disease was not used as a candidate predictor, because congenital heart disease with a left-to-right shunt is a recognized cause of group 1 pulmonary arterial hypertension (PAH) and its inclusion could contaminate the non-group 1 definition; the residual possibility that administrative coding does not fully separate group 1 PAH from SPH is addressed as a limitation.

Candidate predictors were specified a priori based on the existing literature regarding pulmonary hypertension and its associated comorbidities, clinical plausibility, and data availability within the NIS dataset. Comorbidities were identified through ICD-10-CM diagnosis codes utilizing the standard Agency for Healthcare Research and Quality (AHRQ) Clinical Classifications groupings. The 23 selected variables encompassed demographic and socioeconomic factors (age group, race, and median household income quartile), lifestyle-related factors (tobacco use, drug abuse, and alcohol abuse), and various comorbid conditions, including complicated and uncomplicated hypertension, diabetes with and without chronic complications, hyperlipidemia, obesity, peripheral vascular disease, history of myocardial infarction (MI) with revascularization, chronic pulmonary disease, chronic kidney disease, hypothyroidism and other thyroid disorders, prior venous thromboembolism, cancer, arthropathies, depression, and acquired immunodeficiency syndrome.

Predictive modeling was conducted utilizing an Artificial Neural Network (ANN). ANNs are capable of modeling complex, non-linear interactions among variables and handling mixed data types, including both numeric and categorical data, rendering them particularly suitable for large administrative datasets. However, they are less interpretable than traditional regression techniques, and their advantages over logistic regression are highly context-dependent. In the present analysis, a direct comparison with a multivariable logistic regression model was not performed, which is acknowledged as a limitation. The classifier was implemented as a multilayer perceptron (MLP) employing IBM SPSS Statistics version 25.0 (IBM Corp., Armonk, NY, USA). The input layer consisted of 53 units representing 23 candidate variables spanning demographic, socioeconomic, lifestyle, and comorbidity factors after categorical coding in SPSS. Multi-level variables, such as age group, race, and income quartile, expanded into multiple units each. The network comprised a single hidden layer with nine units employing a hyperbolic tangent activation function, and an output layer with two units corresponding to the presence or absence of a coded SPH diagnosis, utilizing a softmax activation function and a cross-entropy error function. The model was trained using the SPSS MLP algorithm with a split of 70% for training and 30% for testing; no balanced sampling was employed. The 17,236,228 hospitalizations reported represent the weighted national estimate from the NIS, whereas the ANN was fitted to the unweighted discharge-level analytic sample. Out of 3,447,246 unweighted female discharge records, 127,703 were excluded due to missing values in one or more model variables, resulting in 3,319,543 valid records. Of these, 2,323,696 (70%) were allocated to the training set, and 995,847 (30%) to the testing set. Model performance was assessed by the percentage of incorrect predictions on the training and testing sets, as well as by the area under the receiver operating characteristic curve (AUC); relative predictor contributions were summarized using normalized variable importance measures. Only internal validation was conducted. Classification performance was further summarized, from the confusion matrix at the default 0.5 probability threshold, as overall accuracy, sensitivity, specificity, positive predictive value (PPV), and negative predictive value (NPV), alongside the AUC. The distribution of predicted pseudo-probabilities by observed outcome was examined graphically to assess discrimination. Institutional Review Board approval was not required for this study, as it utilized a publicly available, de-identified database (NIS). Informed consent was not obtained owing to the retrospective nature of the study and the absence of identifiable patient information.

## 3. Results

Of 17,236,228 weighted female hospitalizations, 648,190 (3.8%) were diagnosed with SPH. Compared to those without SPH, these individuals were more frequently white (67.7% vs. 65.5%) and Black (20.5% vs. 15.5%), and tended to be older (aged ≥65 years: 74.8% versus 40.3%; median age 75 versus 58 years). In-hospital all-cause mortality was higher within the SPH cohort (4.5% versus 1.8%), which also exhibited a greater burden of comorbidities, including complicated hypertension (HTN) (50.6% versus 17.8%), diabetes mellitus (DM) with chronic complications (30.6% versus 13.7%), hyperlipidemia (50.8% versus 29.2%), chronic kidney disease (CKD) (43.3% versus 14.0%), and chronic obstructive pulmonary disease (COPD) (43.4% versus 20.2%). All differences observed were statistically significant, *p* < 0.001. (Table 1).

**Figure 1:**
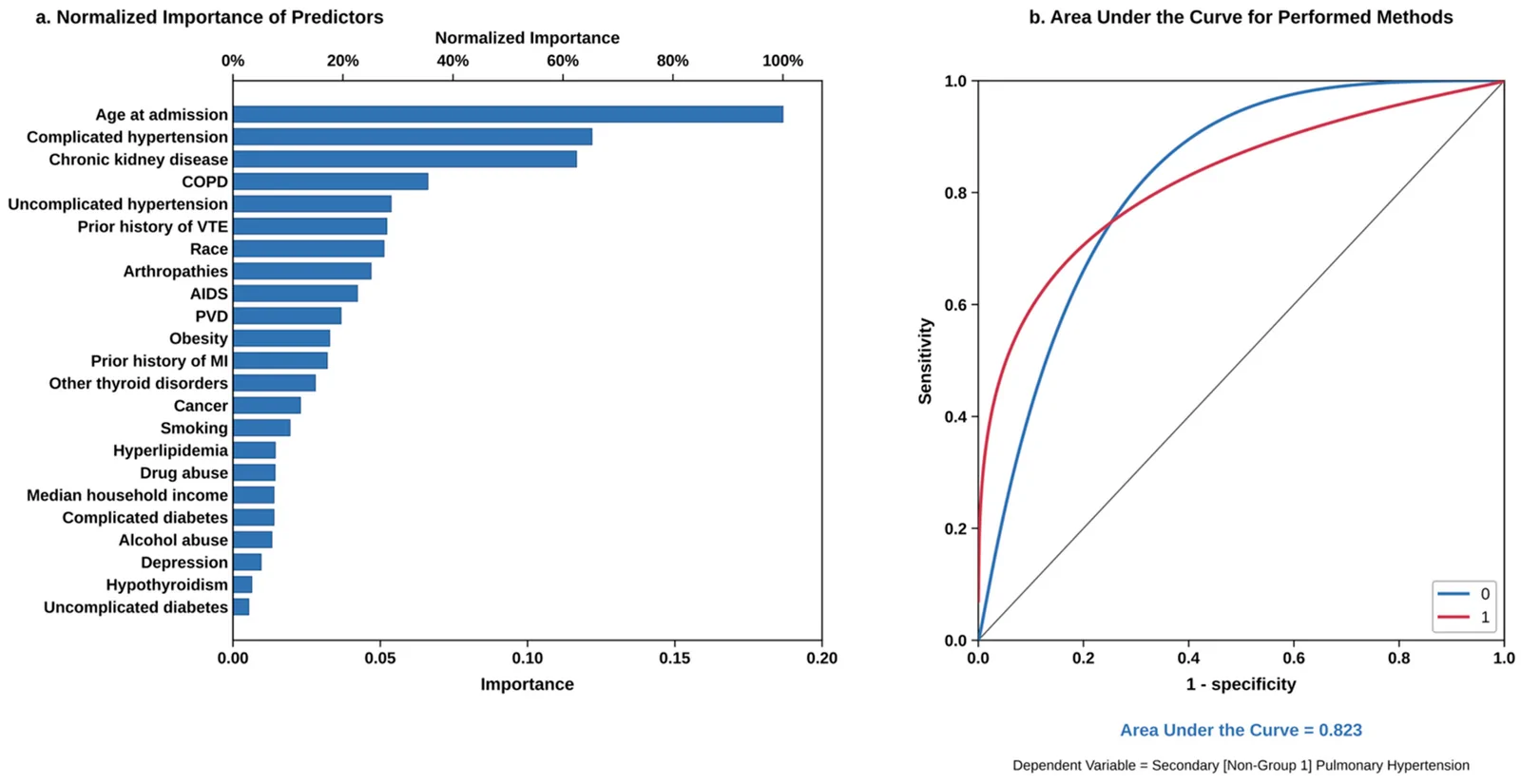
Prediction of SPH in Hospitalized Females by Artificial Neural Network Analysis: Normalized Importance of Predictors & Area Under the Curve for Performed Methods.

The ANN classified a coded SPH diagnosis with an error rate of 3.8% across both datasets and an AUC of 0.823, indicative of good discrimination (Figure 1). Predictors are presented in descending order of normalized importance; for clarity, variables with a normalized importance exceeding 20%, constituting at least one-fifth of the importance of the most influential variable) are emphasized as the primary contributors. The complete ranking is provided subsequently. The principal factors were age at admission (100%), complicated HTN (65.3%), CKD (62.4%), COPD (35.4%), uncomplicated HTN (28.7%), previous venous thromboembolism (VTE) (28.0%), race (27.5%), arthropathies (25.1%), and Acquired Immunodeficiency Syndrome (AIDS) (22.6%). Additional contributors with normalized importance below 20% included peripheral vascular disease (19.6%), obesity (17.6%), prior MI (17.1%), other thyroid disorders (15.0%), cancer (12.3%), smoking (10.3%), hyperlipidemia (7.7%), drug abuse (7.6%), median household income (7.5%), complicated DM (7.4%), alcohol abuse (7.1%), depression (5.1%), hypothyroidism (3.4%), and diabetes without chronic complications (2.9%) (Supplementary Materials).

**Figure 2:**
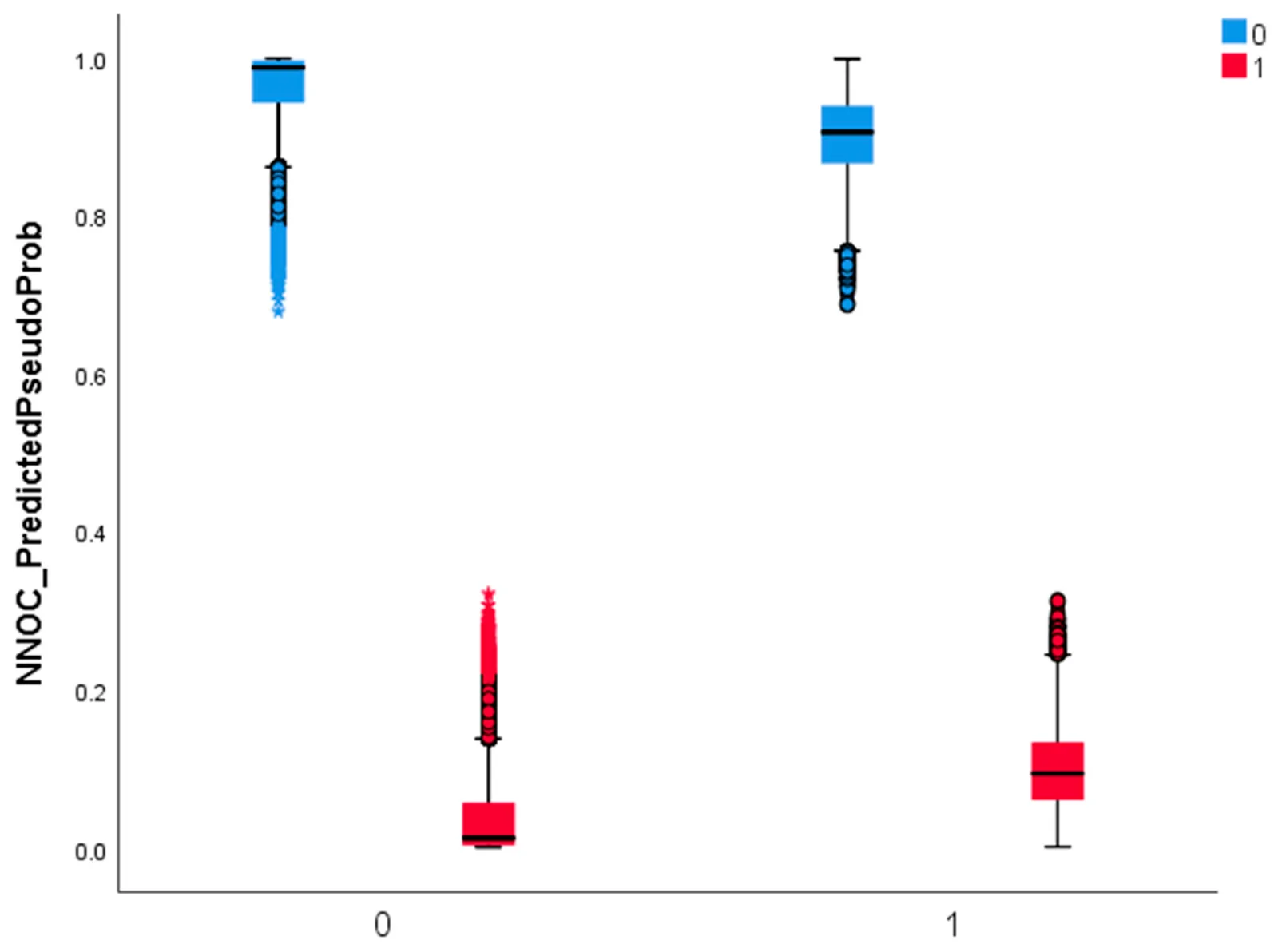
Distribution of ANN-predicted pseudo-probabilities by observed SPH status (0 = no SPH, 1 = SPH). Blue = predicted probability of the non-SPH class; red = predicted probability of the SPH class. Predicted probabilities for the SPH class were higher among women with SPH (median ≈ 0.10, maximum ≈ 0.30) than among women without SPH (median ≈ 0.03) but remained below the 0.5 classification threshold, so no cases were classified as SPH at that threshold.

Classification performance is summarized in Table 2. Overall accuracy was 96.2% in both partitions, with an AUC of 0.823. At the default 0.5 probability threshold, however, the network assigned all patients to the non-SPH class, yielding 100% specificity but 0% sensitivity; PPV was therefore not estimable and NPV was 96.2%. This behavior reflects the marked class imbalance (SPH ≈ 3.8%), and overall accuracy is consequently not an informative measure of performance. The distribution of predicted pseudo-probabilities (Figure 2) nonetheless indicates discrimination between groups: among women without SPH the predicted probability of SPH was very low (median ≈ 0.03), whereas among women with SPH it was higher (median ≈ 0.10, maximum ≈ 0.30). Because these probabilities remained below the 0.5 cutoff, no cases were classified as SPH at the default threshold; a lower operating threshold would be required to recover sensitivity, at the expense of specificity.

## 4. Discussion

Our study shows that secondary pulmonary hypertension (SPH) is more prevalent among older females (median age 75 vs. 58 years), predominantly within White and Black populations. A consistent female predominance in pulmonary hypertension (PH) has been observed over several decades; however, the underlying mechanisms remain inadequately understood. Most proposed explanations are derived from studies of group 1 PAH rather than from administrative SPH cohorts such as ours. Several hypotheses, which are not mutually exclusive, have been proposed. Firstly, sex hormones, particularly estrogen, are strongly implicated: women exhibit greater susceptibility to pulmonary vascular disease, and abnormal estrogen synthesis, metabolism, and signaling are believed to underpin this sexual dimorphism, with estrogen and its metabolites influencing both disease susceptibility and pulmonary vasculature. Estrogen appears to have dual, tissue-specific effects—associated with increased susceptibility in patients yet protective effects in preclinical models—and other sex hormones such as testosterone, progesterone, and dehydroepiandrosterone, along with non-hormonal factors like sex chromosomes and epigenetic modifications, may also contribute. Secondly, the female predominance observed in autoimmune and connective-tissue diseases—which are themselves associated with pulmonary vascular disease—along with fetal microchimerism (the persistence of fetal cells in the maternal circulation, implicated in autoimmune vascular injury), may heighten susceptibility to SPH [4]. Thirdly, women may be exposed to factors less commonly encountered by men, such as anorexigens and exogenous estrogens [5]. Fourthly, advancing age in women correlates with increased vascular and ventricular stiffness, reduced pulmonary artery compliance, and higher pulmonary vascular resistance, potentially promoting pulmonary hypertension.

These observations must be reconciled with apparently divergent findings regarding sex and outcomes. Population-level surveillance indicates that women bear a greater absolute burden of PH-related hospitalizations and mortality [2], reflecting their higher prevalence of PH. Conversely, within diagnosed pulmonary arterial hypertension cohorts, male sex is associated with poorer survival, often referred to as the ‘estrogen paradox’ as reported by Ventetuolo et al. [6] and in more recent reviews [7,8]. These findings are not mutually exclusive; rather, they describe different metrics (population burden versus case-fatality rates) across distinct populations (all-cause or secondary PH versus group 1 PAH). Our administrative data capture the former, which demonstrates a higher prevalence and absolute mortality among hospitalized women with SPH and do not address sex-specific case-fatality rates, as a male comparison group was not included in the analysis.

Several of the most significant correlates of a coded SPH diagnosis, including older age, CKD, COPD, and complicated hypertension, are also indicative of general markers such as multimorbidity, frailty, and increased healthcare utilization. Consequently, the model may mainly reflect an older, multimorbid inpatient phenotype rather than SPH-specific biological factors, and the observed associations should be regarded as characteristics of hospitalized women with an SPH code, rather than as causal predictors of disease.

Compared with the non-SPH cohort, women with SPH demonstrated a higher prevalence of atherosclerotic cardiovascular disease (ASCVD) risk factors, including hypertension, hyperlipidemia, obesity, and diabetes, as well as of established atherosclerotic disease and its sequelae, such as prior myocardial infarction, previous revascularization procedures, and peripheral vascular disease. It is noteworthy that prior myocardial infarction and revascularization indicate established cardiovascular disease or major adverse cardiovascular events rather than risk factors themselves.

These findings are consistent with previous research indicating that patients with SPH, whether related to obstructive sleep apnea [9] or to left heart disease [10], exhibit a higher prevalence of ASCVD risk factors than control groups.

Furthermore, diabetes may contribute to SPH by affecting right ventricular function, analogous to insulin resistance in the left ventricle [11]. Obesity, more prevalent in women, is a known contributor to microvascular dysfunction and to PH through obstructive sleep apnea (OSA), obesity hypoventilation syndrome (group 2 PH), and cardiomyopathy (group 3 PH) [12]. Other comorbidities, including COPD, CKD, arthropathies, cancer, and prior VTE, were more frequently observed in the SPH group. Chronic hypoxic pulmonary vasoconstriction in COPD-related pulmonary hypertension induces fibromuscular intimal thickening and medial smooth muscle proliferation within the pulmonary arterioles, characterized by elevated vasoconstrictors such as endothelin-1 and decreased vasodilators, including endothelial nitric oxide synthase and prostacyclin synthase. Histological remodeling resulting from incomplete resolution of previous venous thromboembolism accounts for the higher incidence of prior VTE in the SPH cohort [13]. The increased prevalence of CKD may reflect volume overload, arteriovenous fistulas, sleep-disordered breathing, dialysis membranes, endothelial dysfunction, vascular calcification, and anemia [14]. The association with arthropathies aligns with the established connection between connective tissue and inflammatory rheumatologic diseases and pulmonary vascular remodeling.

Hospitalized females with SPH also exhibited elevated mortality. This is consistent with prior studies reporting higher rates of PH-related death and hospitalization in women compared to men, with hospitalization frequencies 1.3 to 1.6 times greater [15]. Furthermore, decade-long research conducted by Lakshmanan et al. demonstrated increased mortality among women with PH [16]. These consistent results suggest that the heightened burden of risk factors and comorbidities within the SPH cohort may contribute to these outcomes. As the current analysis relies on administrative data, the mechanistic hypotheses, namely hormonal, autoimmune, and environmental factors, cannot be empirically tested within this study. Instead, they are provided as contextual references derived from the broader scholarly literature, rather than direct results of this study.

The intended use of these findings is hypothesis-generating. The ANN identifies hospitalized women who have a coded SPH diagnosis and, following external and prospective validation, could aid in developing personalized risk-stratification tools or in identifying high-comorbidity inpatients for closer examination. Presently, it is not a validated screening instrument nor a clinically actionable risk calculator, and an AUC of 0.823, while promising, does not independently establish clinical utility. Any translational application must undergo validation in independent, preferably patient-level and prospective datasets, and should be benchmarked against traditional regression models.

An analysis of ClinicalTrials.gov found that women constituted less than 40% of participants in research related to heart disease and stroke [17]. Although PH trials exhibited the highest proportion of female participants (76.3%), these studies predominantly concentrate on disease pathophysiology rather than management strategies [17], thereby contributing to the absence of sex-specific treatment guidance. In a national referral cohort with chronic thromboembolic pulmonary hypertension, women were more likely than men to receive medical (vasodilator) therapy rather than surgical intervention over time [18]. Future research should aim to more comprehensively characterize sex-specific cardiopulmonary health in PH and the related disparities in outcomes and treatment response, thereby facilitating tailored management—an essential objective of personalized medicine. Regulators and researchers should work to identify and eliminate barriers to women’s participation in clinical trials in order to mitigate these disparities.

This study possesses several limitations. It utilized administrative NIS data, which are susceptible to coding and confounding biases and depend on encounter-level rather than patient-level records. Additionally, it examined only a subset of NIS variables, excluding medications, laboratory results, and imaging data. Several further limitations merit emphasis. First, SPH was defined solely by the I27.2x code; however, this code has not been formally validated for SPH, and administrative coding may be prone to misclassification. The I27.2x grouping is heterogeneous, encompassing pulmonary hypertension (PH) groups 2–5, and the NIS does not reliably differentiate these subgroups, thereby preventing SPH from being treated as a singular, uniform entity, which could substantially influence the interpretation of the findings. Second, congenital heart disease with a left-to-right shunt can result in group 1 PAH; although congenital heart disease was not used as a predictor in this study, administrative coding cannot ensure the exclusion of all group 1 patients. Third, the study is cross-sectional, identifying associations based on coded diagnoses at the encounter level; it does not establish temporality, causality, or future hospitalization risk. Fourth, the model underwent only internal validation through a single train–test split and was not compared with logistic regression; external and prospective validation, as well as model comparison, are necessary. Finally, because SPH is more prevalent among females, only female admissions were analyzed, precluding comparative analysis with males. Finally, the marked class imbalance (SPH ≈ 3.8%) means overall accuracy is dominated by the majority class and is not informative, and at the default 0.5 threshold the model classified all patients as non-SPH (sensitivity 0%, specificity 100%). Although the model discriminated risk (AUC 0.823; Figure 2), threshold recalibration—and ideally resampling or cost-sensitive learning—together with external and prospective validation would be required before any classification use.

## 5. Conclusions

Our study demonstrates the potential of ANN models to identify demographic and comorbidity factors associated with a coded SPH diagnosis among hospitalized females. Since the model classifies an existing diagnosis rather than predicting future hospitalization and was only subjected to internal validation, its performance should be interpreted with caution and verified through external, prospective, and ideally patient-level datasets, including benchmarking against conventional regression methods. SPH predominantly affects older females and correlates with a higher burden of cardiovascular risk factors and comorbidities. Upon validation, these findings could facilitate personalized, sex-specific risk stratification and earlier detection of high-risk women, aligning with a personalized medicine approach. Further prospective research is required to confirm these findings and to examine the applicability of ANN models across diverse populations, with particular attention to underrepresented patient groups in clinical trials and randomized studies.

## Figures and Tables

**Table 1:** Baseline Characteristics of Female Hospitalizations with vs. without Secondary Pulmonary Hypertension: Analysis of National Inpatient Sample, 2019.

Variable		Secondary Pulmonary Hypertension (n = 17,236,228)		
		Yes (n = 648,190)	No (n = 16,588,038)	*p*-Value
Age (years) at admission		75	58	<0.001
Race	White	67.7%	65.5%	<0.001
	Black	20.5%	15.5%	
	Hispanic	7.0%	12.1%	
	Asian or Pacific Islander	2.1%	3.2%	
	Native American	0.5%	0.7%	
	Others	2.1%	3.0%	
Median household income national quartile for patient zip code	0–25th	30.9%	30.4%	<0.001
	26–50th	25.8%	25.4%	
	51–75th	24.3%	24.4%	
	76–100th	19.0%	19.8%	
Payer type	Medicare	78.6%	44.0%	<0.001
	Medicaid	8.6%	20.7%	
	Private including HMO	10.5%	29.4%	
	Self-pay	1.3%	3.5%	
	No charge	0.1%	0.3%	
Hospital locations & Teaching Status	Rural	8.1%	9.0%	<0.001
	Urban Nonteaching	17.6%	18.5%	
	Urban Teaching	74.2%	72.5%	
Hospital region	Northeast	18.7%	18.0%	
	Midwest	25.5%	22.1%	
	South	37.1%	40.3%	
	West	18.8%	19.6%	
Hospital admission	Non-Elective	91.4%	73.5%	<0.001
	Elective	8.6%	26.5%	
**Comorbidities**				
Acquired immunodeficiency syndrome (AIDS)		0.43%	0.36%	<0.001
HTN, complicated ^a^		50.6%	17.8%	<0.001
HTN, uncomplicated ^a^		15.4%	29.3%	<0.001
DM, complicated		30.6%	13.7%	<0.001
DM, uncomplicated		9.6%	9.9%	<0.001
Hyperlipidemia		50.8%	29.2%	<0.001
Chronic Kidney Disease		43.3%	14.0%	<0.001
Chronic Pulmonary Disease		43.4%	20.2%	<0.001
Obesity		28.4%	18.6%	<0.001
Alcohol abuse		1.5%	2.8%	<0.001
Drug abuse		2.9%	4.2%	<0.001
Peripheral Vascular Disease		11.3%	4.5%	<0.001
Prior Myocardial Infarction		9.1%	3.7%	<0.001
Prior Percutaneous Coronary Intervention (PCI)		0.71%	0.28%	<0.001
Prior CABG		7.1%	3.3%	<0.001
Tobacco Use		10.3%	13.3%	<0.001
Hypothyroidism		25.3%	16.1%	<0.001
Other Thyroid Disorders		2.6%	1.5%	<0.001
Prior VTE		9.5%	4.6%	<0.001
Depression		16.6%	14.3%	<0.001
Cancer		6.6%	6.2%	<0.001
Arthropathies ^b^		8.3%	4.2%	<0.001
Prior Radiation		1.8%	1.5%	<0.001
Prior Chemotherapy		1.5%	1.6%	<0.001
**In-hospital Outcomes**				
All-cause Mortality		4.5%	1.8%	<0.001
Disposition of the patient	Routine	38.9%	66.4%	<0.001
	Short-term Hospital	2.5%	1.6%	<0.001
	Other transfers SNF, ICF	28.1%	15.4%	<0.001
	Home health care	25.2%	13.8%	<0.001
LOS (days), median [IQR]		5	3	<0.001
Cost USD, median [IQR]		$46,497	$30,823	

**Abbreviations.** HTN, Hypertension; DM, Diabetes Mellitus; VTE, Venous Thromboembolism; CABG, Coronary Artery Bypass Graft; PCI, Percutaneous Coronary Intervention; SNF, Skilled Nursing Facility; ICF, Intermediate Care Facility; HMO, Health Maintenance Organization; IQR, Interquartile Range; LOS, Length of Stay; USD, United States Dollars. *p*-value < 0.05 indicates statistical significance. ^a^ Complicated hypertension refers to hypertension accompanied by documented end-organ involvement, such as hypertensive heart disease or hypertensive chronic kidney disease. In contrast, uncomplicated hypertension signifies essential hypertension without coded target-organ damage, according to the AHRQ/Elixhauser comorbidity grouping. ^b^ Comorbidities were identified utilizing ICD-10-CM diagnosis codes in conjunction with AHRQ Clinical Classifications groupings. Arthropathies include both inflammatory and non-inflammatory joint disorders within this classification; notably, the inflammatory/connective tissue subset (e.g., rheumatoid arthritis and related conditions) is of particular significance due to its established association with pulmonary vascular disease. AIDS and prior PCI have been incorporated into this table to ensure consistency with the predictor set.

**Table 2:** Confusion matrix and classification performance of the artificial neural network for a coded secondary pulmonary hypertension (SPH) diagnosis, by data partition (default 0.5 predicted-probability threshold).

Metric	Training Set (n = 2,323,696)	Testing Set (n = 995,847)
**Confusion matrix (default 0.5 probability threshold)**		
True negatives (observed no SPH, predicted no SPH)	2,235,754	958,390
False positives (observed no SPH, predicted SPH)	0	0
False negatives (observed SPH, predicted no SPH)	87,942	37,457
True positives (observed SPH, predicted SPH)	0	0
**Classification performance**		
Overall accuracy	96.22%	96.24%
Area under the ROC curve (AUC)	0.823	0.823
Sensitivity	0.0%	0.0%
Specificity	100.0%	100.0%
Positive predictive value (PPV)	Not estimable ^a^	Not estimable ^a^
Negative predictive value (NPV)	96.22%	96.24%

AUC, area under the receiver operating characteristic curve; PPV, positive predictive value; NPV, negative predictive value. All metrics are reported at the default 0.5 predicted-probability threshold. Owing to the marked class imbalance (SPH ≈ 3.8%), overall accuracy is dominated by the majority class and is not an informative measure of model performance; discrimination is better reflected by the AUC and by the distribution of predicted probabilities (Figure 2). ^a^ PPV is not estimable because no cases were classified as SPH at this threshold (true positives + false positives = 0).

## Data Availability

The data analyzed in this study are publicly available from the Healthcare Cost and Utilization Project (HCUP) National Inpatient Sample (NIS) 2019 database. Access to the dataset can be obtained through the Agency for Healthcare Research and Quality website (https://hcup-us.ahrq.gov/) after completing the required data use agreement. No new data were generated in this study.

## Supplementary Materials

The following supporting information can be downloaded at: https://www.mdpi.com/article/10.3390/jpm16080421/s1.

## Abbreviations

SPH	Secondary Pulmonary Hypertension
ANN	Artificial Neural Network
AUC	Area Under the Receiver Operating Characteristic Curve
ASCVD	Atherosclerotic Cardiovascular Disease
PH	Pulmonary Hypertension
mPAP	Mean Pulmonary Arterial Pressure
ESC/ERS	European Society of Cardiology/European Respiratory Society
PAP	Pulmonary Arterial Pressure
NIS	National Inpatient Sample
ICD-10-CM	International Classification of Diseases, 10th Revision, Clinical Modification
PAH	Pulmonary Arterial Hypertension
AHRQ	Agency for Healthcare Research and Quality
MI	Myocardial Infarction

## References

[1] Humbert M., Kovacs G., Hoeper M.M., Badagliacca R., Berger R.M.F., Brida M., Carlsen J., Coats A.J.S., Escribano-Subias P., Ferrari P. (2022). 2022 ESC/ERS Guidelines for the Diagnosis and Treatment of Pulmonary Hypertension. Eur. Heart J..

[2] George M.G., Schieb L.J., Ayala C., Talwalkar A., Levant S. (2014). Pulmonary Hypertension Surveillance: United States, 2001 to 2010. Chest.

[3] HCUP-US Databases. https://hcup-us.ahrq.gov/databases.jsp.

[4] Nelson J.L., Lambert N.C. (2025). The When, What, and Where of Naturally-Acquired Microchimerism. Semin. Immunopathol..

[5] White R.J. (2016). Estrogen: Friend or Foe in Pulmonary Hypertension?. Am. J. Respir. Crit. Care Med..

[6] Ventetuolo C.E., Praestgaard A., Palevsky H.I., Klinger J.R., Halpern S.D., Kawut S.M. (2014). Sex and Haemodynamics in Pulmonary Arterial Hypertension. Eur. Respir. J..

[7] Dignam J.P., Sharma S., Stasinopoulos I., MacLean M.R. (2024). Pulmonary Arterial Hypertension: Sex Matters. Br. J. Pharmacol..

[8] Sun Y., Sangam S., Guo Q., Wang J., Tang H., Black S.M., Desai A.A. (2021). Sex Differences, Estrogen Metabolism and Signaling in the Development of Pulmonary Arterial Hypertension. Front. Cardiovasc. Med..

[9] Duan A., Huang Z., Hu M., Zhao Z., Zhao Q., Jin Q., Yan L., Zhang Y., Li X., An C. (2023). The Comorbidity Burden and Disease Phenotype in Pre-Capillary Pulmonary Hypertension: The Contributing Role of Obstructive Sleep Apnea. Sleep Med..

[10] Robbins I.M., Hemnes A.R., Pugh M.E., Brittain E.L., Zhao D.X., Piana R.N., Fong P.P., Newman J.H. (2014). High Prevalence of Occult Pulmonary Venous Hypertension Revealed by Fluid Challenge in Pulmonary Hypertension. Circ. Heart Fail..

[11] Abernethy A.D., Stackhouse K., Hart S., Devendra G., Bashore T.M., Dweik R., Krasuski R.A. (2015). Impact of Diabetes in Patients with Pulmonary Hypertension. Pulm. Circ..

[12] Weatherald J., Huertas A., Boucly A., Guignabert C., Taniguchi Y., Adir Y., Jevnikar M., Savale L., Jaïs X., Peng M. (2018). Association Between BMI and Obesity with Survival in Pulmonary Arterial Hypertension. Chest.

[13] Rodriguez-Arias J.J., García-Álvarez A. (2021). Sex Differences in Pulmonary Hypertension. Front. Aging.

[14] Zhang Q., Wang L., Zeng H., Lv Y., Huang Y. (2018). Epidemiology and Risk Factors in CKD Patients with Pulmonary Hypertension: A Retrospective Study. BMC Nephrol..

[15] Mehari A., Valle O., Gillum R.F. (2014). Trends in Pulmonary Hypertension Mortality and Morbidity. Pulm. Med..

[16] Lakshmanan S., Jankowich M., Wu W.-C., Blackshear C., Abbasi S., Choudhary G. (2020). Gender Differences in Risk Factors Associated with Pulmonary Artery Systolic Pressure, Heart Failure, and Mortality in Blacks: Jackson Heart Study. J. Am. Heart Assoc..

[17] Women Still Underrepresented in Clinical Research, Science and Medicine That Could Save Them from Their No. 1 Killer. https://newsroom.heart.org/news/women-still-underrepresented-in-clinical-research-science-and-medicine-that-could-save-them-from-their-no-1-killer.

[18] Cruz-Utrilla A., Cristo-Ropero M.J., Calderón-Flores M., Velázquez M., López-Gude M.J., Revilla Ostolaza Y., Pérez Vela J.L., de la Cruz-Bértolo J., Bueno H., Arribas Ynsaurriaga F. (2021). Sex Differences in Chronic Thromboembolic Pulmonary Hypertension. Treatment Options over Time in a National Referral Center. J. Clin. Med..

